# The FAM111A Gene: Genetic, Epigenetic, and Pharmacological Targets and Mechanistic Insights with Clinical Relevance

**DOI:** 10.3390/ph19030375

**Published:** 2026-02-27

**Authors:** Kyriaki Hatziagapiou, Feneli Karachaliou, Trias Thireou, Eleni Koniari, Dimitrios Chaniotis, Apostolos Beloukas, Galateia Stathori, Panagiota Kafkaloudi, Bettina Krumbholz, George P. Chrousos, Louis Papageorgiou

**Affiliations:** 1 University Research Institute of Maternal and Child Health and Precision Medicine, School of Medicine, National Kapodistrian University of Athens, 11527 Athens, Greece; hkoniari@med.uoa.gr (E.K.);; 2 Clinical and Translational Research Endocrine Unit, School of Medicine, National Kapodistrian University of Athens, 11528 Athens, Greece; 3 Primary Healthcare Center of Amarousion, 15124 Athens, Greece; p.kafkaloudi@gmail.com (P.K.); dr.b.krum@gmail.com (B.K.); 4 Unit of Pediatric Endocrinology, Metabolism and Diabetes, 3rd Department of Pediatrics, School of Medicine, National Kapodistrian University of Athens, 11528 Athens, Greece; fenkar1@hotmail.com; 5 Laboratory of Genetics, Department of Biotechnology, Agricultural University of Athens, 11855 Athens, Greece; thireou@aua.gr; 6 Department of Biomedical Sciences, University of West Attica, Agioy Spyridonos 28, 12243 Egaleo, Greece; dchaniotis@uniwa.gr (D.C.);

**Keywords:** serine endopeptidases, FAM111A, serine protease domain, catalytic triad, conserved protein motifs, Kenny–Caffey syndrome type 2, osteocraniostenosis, DNA replication stress, replication-fork protection, antiviral restriction factors

## Abstract

**Background/Objectives:** *FAM111A* is a trypsin-like serine protease that has emerged as a regulator of DNA replication, and is directly related to genome stability, protein homeostasis, antiviral defense and cancer progression. Pathogenic variants in *FAM111A* are correlated with genetic syndromes such as Kenny–Caffey syndrome type 2 (KCS2) and gracile bone dysplasia/osteocraniostenosis (GCLEB/OCS). This study focuses on the evolutionary, genetic, and structural analysis of *FAM111A*, in order to identify key regions and candidate pharmacological targets that are related to this enzyme’s function. **Methods:** The methodology of this in silico study includes separate analyses at the sequence, structural and functional levels. Initially, data mining was carried out using NCBI/Protein (2025), and then data filtering was performed in order to identify representative FAM111A sequences for several species. Sequence analysis was then executed through multiple alignments and phylogenetic analyses. Through this, conserved domains and motifs were identified. For structural analysis, human pathogenic mutations and protein structures were identified through searches in biological databases including PDB and ClinVar, and then all data were analyzed in order to identify candidate pharmacological targets related to *FAM111A* function. **Results:** Approximately 1850 FAM111A protein sequences were retrieved for several species, and after filtering processes a dataset of 85 representative sequences was generated. Evolutionary analysis indicates that FAM111A originated in early metazoans, with progressive domain specialization leading to mammal-restricted acquisition of regulatory elements, including the PIP-box PCNA (proliferating cell nuclear antigen) interacting peptide and UBL (ubiquitin-like) domains. The ubiquitin-like/DNA binding domain and catalytic serine protease domain (SPD) are the most conserved, containing seven highly conserved motifs. The structural analysis was based on two protein structures and 34 critical mutations that accumulate in two distinct regions. Finally, by combining the results, six pharmacological targets and 100 inhibitors are proposed. **Conclusions:** Advancing the structural and function characterization of FAM111A, coupled with pharmacological target identification and evolutionary insights, will be critical to validate this underexplored protease as a therapeutic genetic target in genetic disorders, cancer, and antiviral responses.

## 1. Introduction

Proteases are enzymes with indispensable roles in several cellular processes, such as cell proliferation, differentiation, and death [1]. Their function is to cleave amide bonds in protein or peptide substrates in a highly specific and regulated manner, ensuring that substrates are hydrolyzed at the appropriate time and quantity [2]. Since the first attempt to classify proteases based on the proteolysis mechanism and the primary catalytic residue, they are now typically classified into six groups: asparagine, aspartic, cysteine, glutamic, serine, and threonine proteases or metalloproteases [3]. Serine proteases or serine endopeptidases are a functionally diverse group, found in eukaryotes, prokaryotes, archaea, and viruses, and comprise one-third of proteases in *Homo sapiens.* The name stems from the nucleophilic serine (Ser) which attacks the carbonyl moiety of the substrate peptide bond to form an acyl-enzyme intermediate [4]. Serine peptidases are grouped into two broad categories based on their structure: chymotrypsin/trypsin-like, and subtilisin-like. The MEROPS protease classification system counts 16 clans/superfamilies, further divided into several families, based on catalytic site topology. Each superfamily employs the catalytic triad or dyad in a different protein fold, representing convergent evolution of the catalytic mechanism [5,6]. PA (proteases of mixed nucleophile, superfamily A) is a major clan of serine proteases of which S1 constitute the largest protease group in humans [7]. They contain a conserved catalytic triad (charge relay system), consisting of specific set of residues including histidine (His), aspartate (Asp), and Ser, which are all required to cleave the peptide bonds. Nucleophilicity of the catalytic Ser is typically dependent on the catalytic triad. The catalytic triad is preserved in all superfamilies of serine protease enzymes. The specificity of proteases varies, and is largely determined by the specificity pocket “S_1_” near the catalytic triad of the serine protease. The “S1” pocket exhibits notable differences in size and amino acid composition among proteases [2]. The S1 binds complementary to the substrate residue at the N-terminus to the cleavage site. Binding in the S_1_ pocket positions the adjacent peptide bond at the active site for cleavage. Chymotrypsin’s S1 pocket is a deep hydrophobic domain, which favors binding of bulky, aromatic residues, like tyrosine (Tyr), phenylalanine (Phe) and tryptophan (Trp).

**Figure 1:**
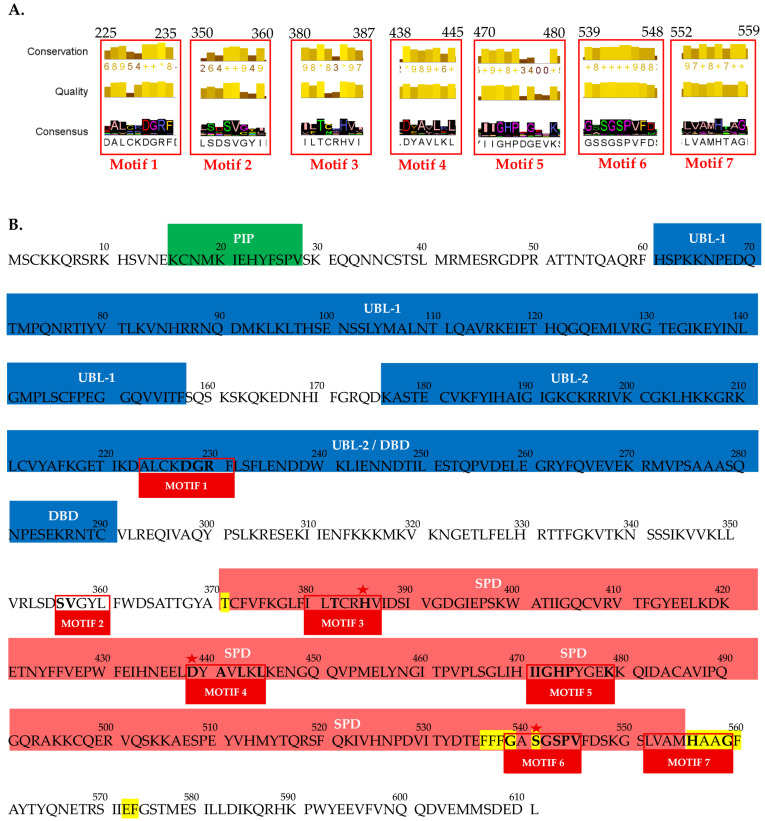
(**A**). The highly conserved motifs as were identified on the consensus sequence based on the MSA. The red boxes indicate the placement of the motifs on the alignment (M1–M7). (**B**). Sequence annotation of the *Homo Sapiens* FAM111A protein (ID: NP_001361777.1) based on structural features and identified conserved motifs. The predicted key amino acid residues that shape the FAM111A S1 pocket positions have been highlighted with yellow and the three common residues of the catalytic side within all serine proteases have been marked with a red star.

**Figure 2:**
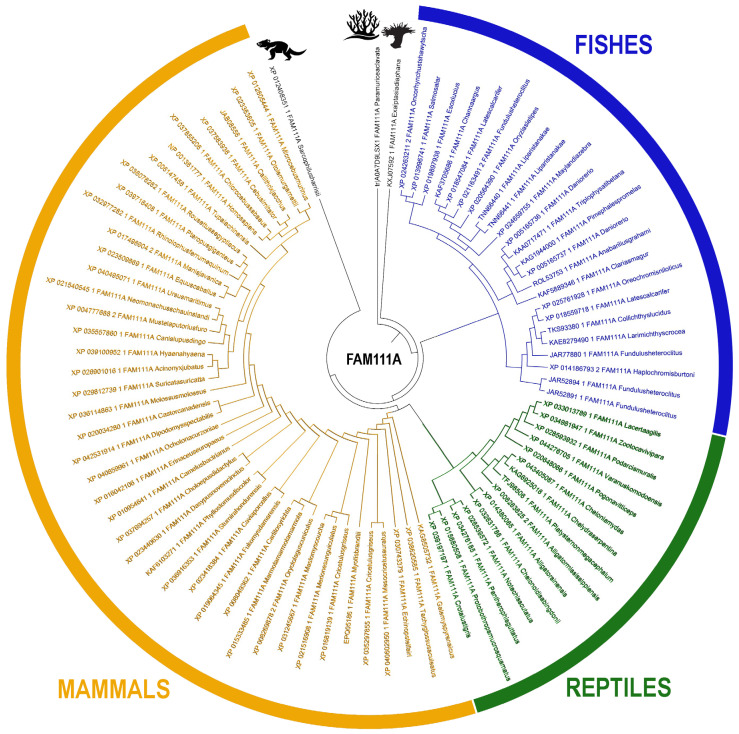
The phylogenetic analysis of the *FAM111A* gene.

*FAM111A* (FAM111 trypsin-like peptidase A/KIAA1895) is mapped on 11q12.1, and encodes a 611-amino acid protein, which functions as a serine protease with homology of its carboxy-terminal to the chymotrypsin/trypsin subfamily of serine proteases [5,6,8]. Chymotrypsin/trypsin-like proteases, such as FA111A, belong to the S1 family of clan PA, and are the most abundant among serine proteases. They are involved in essential physiological processes, including signal transduction, embryonic development, digestion, hemostasis, fibrinolysis, reproduction, immune responses, matrix remodeling, synaptic plasticity, tissue morphogenesis, and regeneration [6]. FAM111A, like other trypsin-like proteases, encompasses four main domains: an N-terminal PCNA ([roliferating cell nuclear antigen) interacting peptide (PIP) box, two ubiquitin-like domains, the UBL-1 and UBL-2/DBD (dsDNA binding domain), and the trypsin 2-like serine protease domain (SPD) at the C-terminus [2,9,10,11,12] (Figure 1 and Figure 2). It is highly similar to its paralog FAM111B with 43% identity in the C-terminal residues [13]. Although S1 family proteases are usually extracellular or membrane-anchored, *FAM111A* is located intracellularly, in the cytoplasmic and nuclear cellular component; however, its functions, and substrates are not completely understood [6,14,15,16]. Currently, there are no experimental or computational approaches that present the complete three-dimensional structure of FAM111A or FAM111B proteins.

*FAM111A* is expressed in all human tissues, including bone and parathyroid gland [2,17]. The highest *FAM111A* expression is found in the adult spleen, thymus, lymph nodes, and tonsils, followed by the adult prostate, small intestine, thyroid gland, lung, kidney, stomach, adrenal glands, liver, and parathyroid glands, and fetal liver. It is suggested to have a crucial role in intracellular pathways regulating skeletal development, and height gain, parathyroid gland development, and parathormone synthesis, thus calcium, and phosphorus homeostasis. FAM111A mRNA and protein levels are cell cycle dependent, and its cellular localization pattern varies during the cell cycle in uninfected cells, and during infection from viruses [18]. In uninfected cells during the G0 quiescent phase it is sequestered predominantly to nucleoli [18]. When cells enter the early G1 phase *FAM111A* is present in nucleoli, but in smaller quantities, whereas during G1/S phase *FAM111A* is barely detectable, and as cells advance to S phase, it is undetectable. Several cellular proteins which are sequestered in nucleoli are relocalized to other cellular compartments, according to the cellular needs. Thus, the low *FAM111A* levels in nucleoli during G1/S, and S phases are likely attributed to protein dispersal, rather reflecting a reduction in overall protein levels, due to degradation or turnover. Contrary, as cells progress towards the G2/M phase of the cell cycle FAM111A levels increase [13,18]. *FAM111A* promoter in G0/quiescent cells is bound by the DREAM, a conserved complex responsible for the coordination of cell cycle-dependent gene expression [13,19,20]. The main function of the DREAM complex is to repress G1/S, and G2/M gene expression during G0, by binding to at least two distinct DNA elements in the promoters of cell cycle-dependent genes [20]. Thus, the expression of DREAM target genes is significantly upregulated in early G1/S or late G2/M phases, compared to G0-arrested cells [13,20,21]. Although *FAM111A* under normal conditions is localized primarily in the nucleus, as a component of the replication fork, it translocates to the cytoplasm in viral-infected cells upon the activation of the cGAS (Cyclic GMP (guanosine monophosphate) -AMP (adenosine monophosphate) synthase)/STING (stimulator of interferon genes) signaling pathway, which is a component of the innate immune system that senses the presence of cytosolic foreign DNA and triggers the transcription if inflammatory genes [10,22,23,24]. FAM111A nuclear export is dependent on its peptidase activity which disrupts the NPC protein NUP62. Mutations of the catalytic triad of FAM111A abolishes its antiviral activity, as its translocation in the cytoplasm requires a functional trypsin-like domain [10].

The main objective of this study is the multidimensional analysis of the biological knowledge related to the FAM111A protein through a bioinformatics pipeline in order to extract novel candidate pharmacological targets related to this important serine protease enzyme. In this direction, an evolutionary analysis was executed in order to extract conclusions regarding protein domains, conserved motifs and key regions withing the identified kingdoms/phylum/species. This information was then annotated in the human FAM111A protein at the sequence, structural and functional levels. Moreover, a specialized structural study was carried out in order to estimate the S1 pocket and catalytic side of the enzyme by combining information from other homologues, and to test a series of candidate inhibitors based on the available space. Finally, the findings were combined with pathogenic mutations related to genetic disorders, and a series of novel proposed pharmacological targets are presented.

## 2. Results

### 2.1. Dataset Collection and Filtering

The initial FAM111A protein dataset contained 1858 entries, related to sequences for multiple species of different phyla. Sequences annotated as “synthetic”, “hypothetical”, “partial”, “low quality”, or “predicted” were removed to eliminate noisy or incomplete data. To reduce redundancy, sequences sharing > 97% sequence identity were further eliminated, retaining only a single representative sequence per taxonomic class. Thus, the final dataset involved 85 FAM111A representative protein sequences; of these, 52% corresponded to mammalian species, 28% to fish, 18% to reptiles and 2% to cnidaria (Supplementary Data S1). Herein, the final set encompassed FAM111A representatives of all phyla of animalia, ranging from simpler organisms such as the *Paramuricea clavate* species of cnidaria, to more complex organisms, including *Homo sapiens*.

### 2.2. Multiple Sequence Analysis and Conserved Motifs

MSA of FAM111A protein sequences was performed to identify evolutionarily conserved regions across representatives of the Animalia kingdom, ranging from cnidarians to mammals. Consistent with known structural features, FAM111A consists of three major conserved regions: PIP box, UBL, and trypsin-like SPD, which were identified in the MSA (Supplementary Data S2 and Figure 1). The MSA revealed two prominent gaps; a large gap in the N-terminal region with a length of approximately 140 residues, and another gap of 90 residues towards the C-terminus, likely corresponding to flexible loops or unstructured regions within FAM111A.

Τhe consensus sequence demonstrated the highest degree of conservation between amino acid positions 220 and 560, across all examined species. These regions correspond to DBD domain, located downstream of UBL-2 and the SPD, indicating that these domains represent the most evolutionarily conserved portions of the protein. In contrast, all the other regions, including UBL1 and PIP displayed strong conservation only among mammalian species and were less conserved within avian and fish lineages. The consensus sequence revealed seven conserved motifs, designated Motif 1 through Motif 7, according to their order in the alignment (Figure 1). Four of the identified highly conserved motifs are located within the SPD, one within the UBL-2/DBD region, one in the region between UBL-2/DBD and SPD, and one at the terminal of the SPD region.

**Figure 3:**
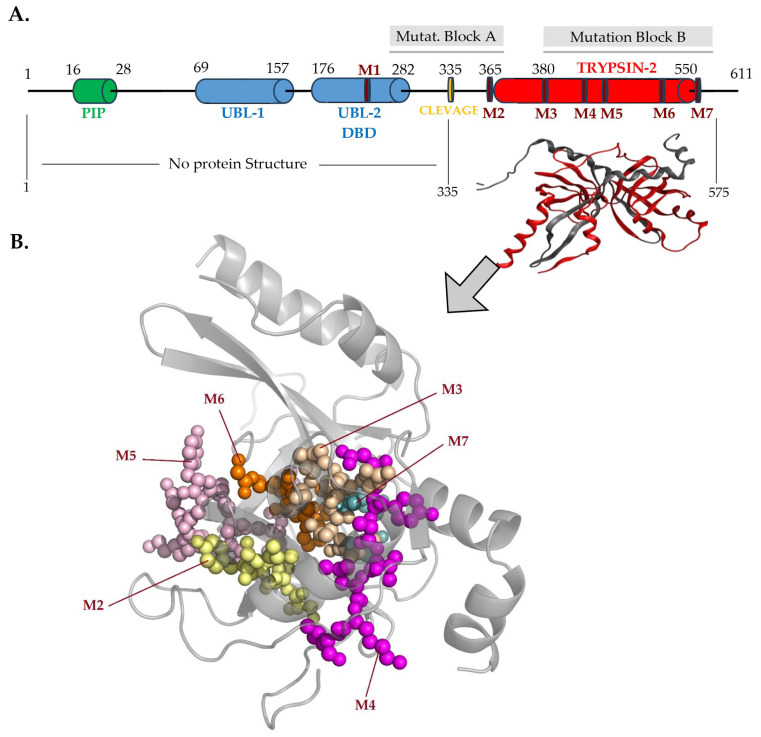
(**A**). Areas of accumulation (Mutation Block A and B) with known pathological mutations in relation to protein domains, conserved motifs and available structures. (**B**). The conserved motifs (M2–M7) as identified in the trypsin-2 domain of the partial crystal structure of FAM111A protein (PDB ID: 8S9K).

The contribution of the conserved amino acids of the motif 1-A [A/T]-[LV]-X-X-D-G-R- [F/L] is identified for the first time and requires further study and investigation for its possible involvement of DNA interactions within the DBD, a feature observed in only a limited number of serine proteases, including FAM111A (Figure 3). Moreover, negatively charged, and conserved key residues Asp228 and Glu229 may be associated with the trypsin S1 pocket, suggesting a possible structural or catalytic relevance [25]. The conserved motif 2-S-V-X-X-[I/L] is also identified for the first time and mapped at the cleavage region of FAM111A. Recent studies indicate that this region forms the α1 helix, which is essential for dimerization of the full-length FAM111A molecule [26].

Additional conserved motifs—motif 3 ([L/V]-[L/F]-T-X-X-H-[V/L]), motif 4 (D-X-A-X-L-X-L), motif 5 (I-I-G-H-P-X-[G/E]-X-X-K), motif 6 (G-X-S-G-S-P-V), and motif 7 ([L/V]-X-X-X-H-X-X-G)—were identified within or adjacent to the SPD. Those conserved motifs are critical for the structural organization and catalytic activity of the FAM111A SPD active site and thus are suggested as potential pharmacological targets. Notably, motifs 3, 4, and 5 encompass the key residues His385, Asp439, and Ser541, which form the catalytic side of SPD. Moreover, the highly conserved residues Gly539 and Ser541 within motif 6 are essential for the formation of the oxyanion hole in the catalytic triad, further emphasizing the functional importance of these motifs in FAM111A enzymatic activity [2,6,17,26,27,28,29].

### 2.3. The Evolution of FAM111A Gene

The evolutionary history of the FAM111A gene indicates an origin from lower taxa species, notably the *Plexauridae* and *Aiptasiidae* families with subsequent retention and diversification through higher taxa of the phylum Chordata, culminating in humans (Figure 2, Supplementary Data S3). Notably, FAM111A orthologues were not detected in any avian genomes examined [2,30,31,32].

Vertebrate classes segregate into well-supported, monophyletic clades, and the most highly conserved regions across taxa correspond to the more ancient functional modules—principally the trypsin-2–like serine protease domain and, secondarily UBL-domains. The trypsin-2–like protease domain appears to be ancestral and is shared both among vertebrates and among several other serine protease families, whereas lineage-restricted features, such as a canonical PIP- box, are observed only in mammals. These patterns of conservation and lineage specificity, as reflected in the distribution of conserved motifs within individual protein domains, provide a mechanistic framework for interpreting how structural innovation and domain accretion have shaped *FAM111A*’s evolution and functionality.

### 2.4. The FAM111A Structure and the Predicted Inhibitors

The FAM111A protein structure, like sequence, contains four distinct functional regions including PIB box and UBL1, UBL2/DBD and SPD domains (Figure 3). The crystal structure of the FAM111A has been partially solved. Only the SPD of this protein (residues 332-600) has been determined by x-ray crystallography (PDBID: 8S9K) in 2023 by Palani et al. [26]. The PIP box (residues 16-28) is a short, partially conserved peptide motif that tethers DNA replication and repair factors to PCNA [33,34]. It is defined as Qxxφxxψψ, in which “Q” refers to glutamine, “φ” to a hydrophobic residue, “ψ” to an aromatic residue, commonly phenylalanine or tyrosine, and “x” to any amino acid [35]. Interactions involving the PIP-box are hydrophobic and are highly favorable in water solvents [33,34,36,37,38,39,40]. UBL-1 and UBL-2 domains with a ubiquitin-like fold structure promote noncovalent protein–protein interactions, especially the 26S proteasome, increasing its degradative capacity of intracellular proteins [41,42]. The UBL-2 domain exhibits a degree of overlap with the ssDNA-binding region of the FAM111A protein and contains the identified conserved motif 1 [17] (Figure 3).

**Figure 4:**
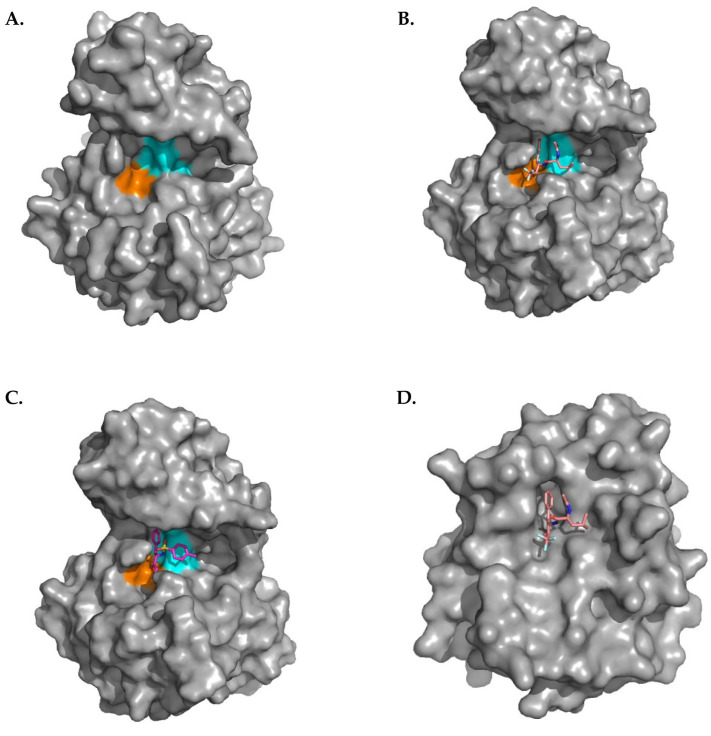
(**A**). The FAM111A-predicted S1 pocket and the identified conserved motifs. Orange and blue colors show the positions occupied by motifs 6 and 7 respectively. (**B**). N-Acetyl-leucyl-phenylalanyl trifluoromethyl ketone docked to FAM111A SPD (PDBID: 8S9K) (**C**). N-p-Tosyl-L-Phe chloromethyl ketone docked to FAM111A (PDBID: 8S9K). (**D**). N-Acetyl-leucyl-phenylalanyl trifluoromethyl ketone located in chymotrypsin (PDBID: 7GCH).

The SPD in C-terminus is the most conserved region within all studied species and is found to be highly conserved among S1 serine proteases [17,28]. This region contains the identified conserved motifs 2–7 (Figure 1 and Figure 3). In order to determine the key residues located in the FAM111A S1 pocket, in addition to the protein structure mentioned (PDBID: 8S9K), the crystal structure of chymotrypsin (PDBID:7GCH) was also used [26,43]. Proteins FAM111A and gamma chymotrypsin share structural similarity with some distinct differences [25,43]. Based on the literature and the alignment of the above structures, key residues that shape the FAM111A S1 pocket include Tyr369, His385, Asp439, Phe536, Phe537, Phe538, Ser541, His556, Ala557, Ala558, Gly559, Phe560, Glu573, Phe574, while the oxyanion hole is delineated by Ser541 and Gly539 (Figure 1 and Figure 4). FAM111A SPD is narrower than chymotrypsin’s and functions by cleaving smaller molecules instead of accommodating whole proteins. The catalytic residues of FAM111A SPD are His385, Asp439, and Ser541. They are placed close to the interaction point of the substrate in the tertiary structure of the protein in the S1 pocket in order to hydrolyze a peptide bond [6]. FAM111A proteolytic activity harbors a specificity for phenylalanine, and to a lesser extent for tyrosine, and tryptophane, similarly to gamma chymotrypsin [26]. Dimerization of the SPD is essential for a proper substrate cleavage function in vivo, by stabilizing the conformation of oxyanion hole at the active site, whereas is dispensable for FAM111A autocleavage activity [26]. The oxyanion hole based on the literature is structurally linked to the catalytic triad [1,6].

Docking analysis of the FAM111A inhibitor N-p-Tosyl-L-Phe chloromethyl ketone and the chymotrypsin inhibitor N-Acetyl-leucyl-phenylalanyl trifluoromethyl ketone was performed in the identified FAM111A S1 pocket. Analysis of the docking results reveals that the identified conserved motifs 6 and 7 are critical for the protein–ligand binding, which is expected to block natural substrates (Figure 4). Last, but not least, using those inhibitors, a list of candidate inhibitors that are sufficiently similar based on three-dimensional structure and stereochemistry is provided for further research and testing (Supplementary Data S4).

### 2.5. Mutations, Functions and Genetic Syndromes

FAM111A heterozygous point mutations are associated with two severe developmental syndromes; Kenny-Caffey syndrome type 2 (KCS2) (OMIM #127000) and gracile bone dysplasia/Osteocraniostenosis (GCLEB/OCS, OMIM #602361) with a lethal phenotype [17,44,45] (Table 1). The majority of them accumulate in two distinct regions including mutation block A and B (Figure 3). KCS2 is associated with one of the well-known pathogenic variants, including the p.P527T, p.E535G, and p.R569H within the mutation block 2 (Table 1) [2,9,10,11,12,28,46]. The latter is the most common pathogenic mutation in KCS2. On the other hand, the most common pathogenic mutations in GCLEB/OCS are p.T338A and p.S342del. Several other mutations that are related to those two syndromes or FAM111A functions are reported in Table 1.

FAM111A demonstrates an intrinsic autocleavage activity that can occur intramolecularly and intermolecularly [28]. The prominent cleavage site is determined between Phe334 and Gly335 [28]. Interestingly, disease-associated mutations including Arg569His, Thr338Ala, and Tyr511His point mutations, implicated in KCS2, and Ser342Del and Asp528Gly in OCS/GCLEB, cause gain-of-function (GOF) in FAM111A activity, whereas no loss-of-function mutations are reported [28]. Thus, the autocleavage activity of FAM111A in vivo in KCS2 and OCS is amplified, rather than hyperactivation of FAM111A’s normal protease function, converting the full-length protein to smaller fragments, leading to functional FAM111A deficiency [17,28]. However, hyper-autocleavage could indirectly result in unregulated FAM111A peptidase function, due to cleavage between the autoinhibitory domain in N-terminal region and the SPD. Also, disease-associated FAM111A GOF mutations may render SPD resistant to the N-terminal autoinhibitory effect [26,47].

FAM111A unrestrained proteolytic activity may cause abnormal degradation of DNA-binding proteins such as RFC1, PCNA, and RPB1 during replication and transcription, leading to cellular apoptosis [11,29]. Although as aforementioned FAM111A promotes DNA replication under basal conditions, constitutive FAM111A peptidase activity, associated with *FAM111A* mutations in KCS2 and OCS, could lead to ssDNA formation in S phase entry [17]. Also, these *FAM111A* mutations may induce cellular apoptosis due to aberrant nuclear morphology, disruption of nuclear pore complexes (NPCs) and pore distribution [10,11,28]. NPCs are implicated in protein and mRNA transportation between the nucleus and the cytoplasm, heterochromatin formation, gene silencing, transcriptional activation, and RNA processing [48]. FAM111A hyperactive protease activity targets NPC factors including the transmembrane NUP (nucleoporin) POM121, NPC cytoplasmic filaments NUP98, and NUP214, the channel NUP NUP62, and the nuclear basket NUPs NUP153, and NUP50 [11,49].

Collectively, the previous mechanisms may contribute to the deleterious symptoms observed in KCS2 and OCS. For example, amplified FAM111A protease activity may be responsible for hypoparathyroidism, and hypocalcemia due to cellular apoptosis, and compromised parathyroid development in embryonic morphogenesis, or increased degradation of molecules implicated in CaSR desensitization and internalization such as *β*-arrestins 1 and 2 and G protein receptor kinases (GRKs). Indeed, KCS2 phenotype resembles CaSR gain-of-function mutations, in which patients exhibit mild to moderate low hypocalcemia with inappropriately low PTH [50]. In LM/Bc mice GOF FAM111A mutations near the autocleavage site such as the mutation Thr335Lys in mice that corresponds to the human Thr338Ala mutation, is associated with hypocalcemia, hypomagnesemia and hypermagnesuria. Also, microphthalmia, and occasional birth of small mice which fail to thrive, are linked with the mutation Thr335Lys [51,52]. However, in other mice models loss of function mutations in SPD, e.g., frameshift insertion (c.1450insA) or large deletion (c.1253-1464del), resulting in premature stop codon at 545 and 472 of FAM111A or Fam111a knockout (FAM111a−/−) are not associated with KCS2 overt phenotype, i.e., hypocalcemia, low serum PTH, abnormal bone mineral density and microarchitecture, suggesting that SPD is dispensable for calcium homeostasis in mice [46,53]. Fam111a−/− mice are viable, and do not exhibit other electrolyte disturbances, e.g., serum, and urine magnesium, phosphorus, sodium, and potassium. Compensatory mechanisms, such as other proteases may compensate for the loss of FAM111A function, protecting against KCS2 symptoms. Also, animal models do not always fully recapitulate human disease [51,53].

*FAM111A* functions as an accessory replication fork component, implicated in PCNA loading during DNA replication, and promoting S-phase entry and DNA synthesis [9,29]. It preferentially binds to intact ssDNA (single stranded DNA), and Y-shaped replication forks, and weakly to dsDNA via its central region (176–282) [28]. The mutation Phe231Ala in motif 1 within the mutation block A decreased DNA binding to ssDNA, indicating the important role of Phe231(Figure 2 and Figure 4) [28]. PCNA is a DNA clamp, serving as a hub for complex protein network. It is a ring-shaped homotrimer that encircles and moves along double-stranded DNA (ds-DNA), serving as a processivity promoting factor for DNA polymerase delta (DNA Pol δ), regulating the access to DNA of several enzymes, and enhancing their catalytic activity [33,34,54,55]. Thus, it is implicated in DNA replication and repair, bypass of DNA damage, chromatin restoration, sister-chromatid cohesion and cell cycle regulation. The aforementioned functions of PCNA are mediated via its ability to recruit with different binding affinities more than 200 proteins to the replication fork, including *FAM111A* via its PIP-box [9,10,34,56,57,58].

*FAM111A* facilitates prior to S-phase entry firing of active and dormant -in response to replicative stress-origins, thus activating DNA replication [17]. Also, *FAM111A* promotes ssDNA formation in response to fork stalling, generated by polymerase–helicase uncoupling or nucleolytic processing, upon exposure to genotoxic challenges, e.g., the reversible, and selective DNA polymerase-*α* inhibitor aphidicolin (APH), and hydroxyurea (HU) [17]. *FAM111A* may regulate transcription, as it interacts with ZNF226 (Zinc finger protein 226), a transcription factor, playing a role in transcriptional repression [59].

Mutations in the *FAM111A* PIP-box (PIPmt) are implicated with diffuse protein pan-nuclear distribution [9]. PIPmt abrogate PCNA loading or maintenance on chromatin and/or its stability, leading to cell cycle arrest outside S phase [29]. Interestingly, the replication cannot be restored with overexpression of PCNA. Similarly, *FAM111A* depletion is also associated with reduced levels of chromatin-bound PCNA, and marked delay in cellular progression from G1 through S phase to G2/M [9,18]. However, *FAM111A* overexpression could also result in cell cycle arrest in early S phase, probably reflecting that PIP box-containing proteins compete with other PCNA binding partners, implicated in DNA replication, e.g., DNA polymerase *δ* or *ε*, DNA ligase I, and FEN-1 (Flap Structure-Specific Endonuclease 1) [9,33,34]. Also, *FAM111A* overexpression might inhibit DNA replication by displacing, via its proteolytic activity, RFC1 (Replication factor C 1) chromatin association; RFC1 serves as a clamp loader around DNA for PCNA in an ATP-dependent manner [29,54,55]. Elevated *FAM111A* expression has an adverse effect on transcription by decreasing via its protease activity chromatin-bound RPB1 (DNA-directed RNA polymerase II subunit), which is the catalytic and largest component of RNA polymerase II. Thus, elevated FAM111A by antagonizing DNA replication and transcription might finally trigger caspase-dependent cellular apoptosis [29].

## 3. Discussion

### 3.1. Proteases, Serine Proteases, and Their Inhibitors

Serpins (SERine Protease INhibitorS) are natural inhibitors which antagonize the activity of serine proteases through a conserved mechanism, comprising of dramatic conformational alterations. All serpins share a core structure comprising of three β-sheets (A, B, C), and eight or nine α- helices (hA–hI), and an exposed flexible reactive center loop (RCL), which are positioned in the middle, and at the C-terminus of the molecule, respectively. RCL contains a substrate-mimicking motif with a scissile bond between residues P1 and P1’ which forms a reversible Michaelis-Menten complex with its target serine protease [7,60,61]. Upon cleavage at P1-P1′ by the docking protease, a conformational change of serpin is triggered, and is refolded into a hyper-stable conformation in which the protease becomes covalently bound to the main chain carbonyl carbon of the P1 residue of the serpin. The serpin traps the covalently linked protease, translocating it to the opposite pole of the molecule, and distorting its catalytic site [61,62,63]. The serpin-protease complex is subsequently eliminated from the circulation via scavenger receptors [7,60,61]. As aforementioned, SPI-1, a member of the serpin family of serine protease inhibitors, preferentially antagonizes FAM111A during poxvirus infection by forming a stable covalent complex of FAM111A SPD with His_6_-T7-SPI-1. The serine residue S541 of the catalytic triad of FAM111A is essential for the complex formation. Interestingly, SPI-1 does not effectively inhibit chymotrypsin and cathepsin G, although they share the classic serine protease active site structure with FAM111A [64]. Investigation of similar serpins and targets of FAM111A may further elucidate FAM111A function, and lead to novel therapeutic strategies for FAM111A-related diseases.

The chymotrypsin-like SPD in the C-terminus domain of FAM111A functions as a serine protease. Although its substrates are largely unknown, long flexible peptide substrates rather than globular proteins preferentially gain access to its narrow active site [47]. An intact FAM111A DNA-binding domain is necessary for its protease activity; thus, it is speculated that FAM111A might be activated when sensing ssDNA, responding to stress imposed by fork collisions with protein obstacles during replication [28]. FAM111A via its protease function has a role in the progression of the DNA replication machinery by mitigating the effect of protein obstacles, such as DNA protein crosslinks (DPCs) [12,28]. The induction of DPCs is a common pharmacological action for anti-cancer drugs, as stalling the replication fork has a rate-limiting effect on cancer cell growth, and proliferation due to dsDNA breaks.

In this study, a series of candidate inhibitors that are potentially able to bind in the S1 pocket of the enzyme are for the first time presented. Similar work has been successfully carried out experimentally in the past by Brady et al. 1990 for another serine protease enzyme, the gamma chymotrypsin which share very similar structural morphology in the SPD and its catalytic side with the FAM111A corresponding region [43].

### 3.2. The FAM111A as a Viral Host Range Restriction Factor

FAM111A serves as a critical host restriction factor against various DNA and RNA viruses, including Simian vacuolating virus 40 (SV40), Adenovirus 5 (Ad5), Vaccinia virus (VACV) and Zika Virus (ZIKV) [18]. During SV40 and Ad5 infections, FAM111A is recruited to viral replication sites where it binds to viral chromatin and the viral Large T antigen (LTag) protein to inhibit viral gene expression, replication and cellular transformation [13,18,64,65,66,67]. It exerts its antiviral effects through the C-terminal half SPD, which can recognize and degrade viral nucleoprotein complexes. This defense mechanism is so potent that SV40 has evolved a countermeasure where the LTag protein binds to and inactivates FAM111A to promote viral assembly and cellular lysis [13,18]. Interestingly, the expression of FAM111A is regulated by host factors like the DREAM complex and interferon regulatory factor 2 (IRF2), the latter of which upregulates FAM111A during ZIKV and VACV infections to bolster the immune response [68]. In the context of poxviruses like VACV, FAM111A operates within an antiviral network alongside interferon regulatory factor 2 (IRF2) and replication factor C3 (RFC3) [68]. When the virus infects a cell, FAM111A localizes near cytoplasmic viral factories and uses its DNA-binding domain to interact with the essential viral I3 protein. FAM111A then facilitates the degradation of I3 via autophagy, effectively arresting viral DNA replication and protein expression [10,64,69,70]. To circumvent this, certain orthopoxviruses utilize a serine protease inhibitor called SPI-1. SPI-1 enters the host nucleus to prevent the nuclear export of FAM111A and inhibits its peptidase activity, thereby protecting the virus from degradation and ensuring successful propagation [10,64]. The results of this work regarding the candidate pharmacological targets described in these important regions including conserved motifs 1, 4, 5, 6 and 7 could be studied for their candidate antiviral activity.

### 3.3. The FAM111A as a Prognostic Biomarker in Cancer

FAM111A’s direct implication in replication fork progression may reveal correlations between FAM111A and tumorigenesis. FAM111A possibly is associated with an immunosuppressive tumor-microenvironment, displaying prominent CD8 T-cell, myeloid dendritic cells and M2 macrophage infiltrates [71,72]. FAM111A expression is a potential independent prognostic biomarker for gliomas, predicting worse survival outcomes. It is significantly up-regulated in grade III anaplastic astrocytomas IDH (Isocitrate dehydrogenase) wild-type, and downregulated in the IDH mutant-1p19q codeletion (IDHmut-codel) oligodendrogliomas [71,73,74,75]. FAM111A overexpression is demonstrated in gastric cancer, and is correlated with poor survival in HCC (hepatocellular carcinoma), and thyroid cancer [72,76]. Long noncoding RNA (lncRNA) of FAM111A [FAM111A-divergent transcript; FAM111A-DT], when undergoing N6-methyladenosine (m6A) epitranscriptomic modifications by m6A methyltransferases to the stable m6A-modified FAM111DT, promotes HCC proliferation, and confers poor differentiation, advanced clinical stage, and poor survival of HCC patients. The m6A-modified FAM111DT interacts with m6A-binding protein YTHDC1 (YTH N6-Methyladenosine RNA Binding Protein C1), and recruits the histone demethylase KDM3B (Histone lysine demethylase 3B) to FAM111A promoter, directing the demethylation of H3K9me2 (dimethylated histone H3 at the 9th lysine residue of heterochromatin), and triggering transcription of wild-type FAM111A [76]. *FAM111A-DT* and *LINC02550* function as oncogenes in thyroid cancer, by activating the NF-κB signaling pathway, and are positively correlated with poor outcome [77,78]. FAM111A mRNA (messenger RNA) is identified as prognostic biomarker in head and neck squamous cell carcinoma (HNSCC) [79]. FAM111A along with other proteases such as FAM111B, CFB (complement factor B; serine protease), PSMB8 (proteasome subunit beta type-8; 20S proteasome subunit beta-5i), PSMB9 (proteasome subunit beta type-9; 20S proteasome subunit beta-1i), CASP7 (caspase-7), and PRSS16 (Thymus-specific serine protease) correlates with cancer progression, invasion, and metastasis in locally advanced cervical cancer [76,80].

FAM111A protects cells from several anti-cancer drugs, especially the cytotoxic activity of TOP1 (topoisomerase I) inhibitors and PARP (poly ADP-ribose [olymerase) [28]. TOP1 inhibitors, such as camptothecin (CPT) bind at the interface of TOP1-DNA complexes, thus selectively trap, and stabilize Top1 cleavage complexes during DNA replication and/or transcription, inducing TOP-DPCs [28,47]. TOP-DPCs disrupt DNA replication, transcription, and recombination, by generating replication-fork collision in dividing cells [81,82,83,84]. PARP inhibitors such as niraparib, and talazoparib induce nucleoprotein complexes by trapping PARP1 non-covalently at DNA during the single-strand breaks repair mechanism. The PARP1-DNA complexes mimic DPCs, block DNA replication and cause DSBs, cell-cycle arrest in G2/M phases, and eventually cell death [28]. FAM111A PIP box tethers its SPD to replication forks, and prevents fork collapse due to protein obstacles, regardless of their DNA cross-link status, i.e., during treatment with TOP1 inhibitors, and PARP inhibitor, by facilitating the in vivo repair of DPCs (TOP1cc accumulation, PARP1-DNA nucleoprotein complexes), via its putative serine protease activity, and releasing DNA replication [17,28]. On the contrary, FAM111A-knockout cells are sensitized in those agents [26,28].

## 4. Materials and Methods

### 4.1. Dataset Collection and Pre-Analysis

A comprehensive search was conducted in the NCBI/Protein database to extract all protein sequences associated with *FAM111A*. Data were filtered using several criteria in header information in order to avoid unrelated or incomplete sequences, and a dataset of representative protein sequences with less than 97% protein similarity was generated. The taxonomic groups of the species resulted were then studied based on the kingdoms/phylum/class in the tree of life, and the protein domains were identified using the information provided by the data and other bioinformatic platforms such as InterPro (InterProScan version: 5.68-100.0) [26,43]. Available corresponding protein structures were obtained from the Protein Data Bank (PDB) and pathogenic and likely pathogenic variants were extracted from the NCBI/ClinVar database and relevant literature. Last, but not least, all the extracted information was then annotated within the *Homo sapiens* FAM111A protein sequence.

### 4.2. MSA, Conserved Motifs and Variants Analysis

Multiple sequence alignment (MSA) was performed using the MATLAB R2020b Bioinformatics Toolbox [31], employing a guide tree and the progressive MSA algorithm as described in previous studies [24,32]. The alignments were visualized in the Jalview platform [85] and conserved motifs were identified via the alignment annotation module. The selected residues of the consensus sequence for a conserved motif demonstrated a Jalview scoring ≥ 10 and a frequency ≥ 90%. A score of 11 denotes absolute conservation, whereas a score of 10 indicates that, despite variation, physicochemical properties are conserved [85]. Thus, conserved motifs of about 10 amino acids that appeared common across all organisms were selected based on MSA. Highly conserved residues within the conserved motifs were annotated according the extracted pathogenic and likely pathogenic mutations.

### 4.3. Phylogenetic Analysis

A phylogenetic tree was constructed in the MATLAB Bioinformatics Toolbox based on the UPGMA algorithm (unweighted pair-group method) consistent with methodologies reported in previous studies [30,31]. The visualization and editing of the constructed phylogenetic tree were performed using the MEGA and iTOL platforms [30,31,32]. The iTOL Annotation Editor for spreadsheets was also used to divide the tree into clusters and assign animalia phyla to the terminal nodes [86]. The topology of the phylogenetic tree was studied to define the clusters of the monophyletic branches. The resulting topology was analyzed to identify monophyletic clusters, which were subsequently correlated with specific taxonomic groups, protein domains, and conserved motifs.

### 4.4. Structural Analysis and Pharmacological Targets Identification

A structural analysis was performed in order to estimate key structural and functional regions based on the identified domains within the FAM111A protein. Protein structures were studied based on secondary structure elements, and in cases where additional substrate data were available, candidate interaction sites were estimated in order to delineate the DNA binding site and the catalytic center of the enzyme. Combining these findings, the extracted conserved motifs were then evaluated for their structural and functional significance. Conserved motifs were categorized based on their localization in key domains or regions of FAM111A, and examined in the context of existing knowledge on serine proteases. Residues representing the most sensitive or functionally critical positions were annotated, including catalytic sites, pathogenic/likely pathogenic mutations, and positions exhibiting high conservation across evolutionary lineages. Physicochemical properties and structural features were then integrated in order to identify the candidate pharmacological targets, and providing insight into residues or regions which may serve as sensitive points for therapeutic intervention. As part of the structural study, the DALI Protein Structure Comparison Server was used to align the FAM111A and the chymotrypsin structures (PDBIDs: 8S9K and 7GCH) [26,43,85]. Docking studies were performed in silico using AutoDock Vina version 1.2.0 [86]. For the ligand-based screening, DrugRep version 1.0 was used with the approved drug library and FitDock parameters [87].

## 5. Conclusions

FAM111A has emerged as a multifunctional serine protease that has roles extending far beyond its initial annotation as a trypsin-like enzyme. Functionally, FAM111A occupies a unique intersection between DNA replication, replication-stress responses, protein obstacle removal, antiviral immunity, and cancer. These dual roles highlight the delicate protease balance required for physiological function and explain why perturbations in FAM111A activity produce such severe multisystem disease phenotypes. The study of the FAM111A, will allow understanding of their crucial genetic, structural and functional characteristics in relation to the large family of proteases. Accordingly, this research will provide the foundation for developing strategies to mitigate pathogenic conditions linked to alterations in the FAM111A, and to engage in important biological pathways associated with a series of candidate pharmacological targets and inhibitors of this group of serine proteases.

## Figures and Tables

**Table 1:** Pathogenic mutation in FAM111A and crucial variants that may affect protein function.

A/A	Mutation	Region	Diseases
1	F231A	PIP	Decreased DNA binding
2	C226F *	UBL2/DBD|Mutation Block 1|Motif 1	Disrupted protein function
3	R230S *	UBL2/DBD|Mutation Block 1|Motif 1	Disrupted protein function
4	E285Q *	UBL2/DBD|Mutation Block 1	OCS
5	L292fs *	UBL2/DBD|Mutation Block 1	KCS2
6	I311F	Mutation Block 1	KCS2|OCS
7	G323E	Mutation Block 1	KCS2
8	L326I	Mutation Block 1	KCS2
9	T338A	Mutation Block 1	KCS2|OCS
10	S342del	Mutation Block 1	KCS2|OCS
11	V357[I/fs] *	Mutation Block 1|Motif 2	Disrupted protein function
12	I380V *	SPD|Mutation Block 2|Motif 3	OCS
13	T382I *	SPD|Mutation Block 2|Motif 3	Disrupted protein function
14	R384W *	SPD|Mutation Block 2|Motif 3	Disrupted protein function
15	H385Y *	SPD|Mutation Block 2|Motif 3	KCS2
16	D439	SPD|Mutation Block 2|Motif 4	KCS2
17	H470Y	SPD|Mutation Block 2|Motif 5	KCS2
18	C485F	SPD|Mutation Block 2	KCS2
19	Y511H	SPD|Mutation Block 2	KCS2
20	M514I	SPD|Mutation Block 2	KCS2|OCS
21	F520C	SPD|Mutation Block 2	KCS2
22	P527T	SPD|Mutation Block 2	KCS2|OCS
23	D528G	SPD|Mutation Block 2	KCS2|OCS
24	E535G	SPD|Mutation Block 2	KCS2
25	S541[Y/P]	SPD|Mutation Block 2|Motif 6	KCS2
26	G542 [S/V/D] *	SPD|Mutation Block 2|Motif 6	Disrupted protein function FAM111A-related disorders
27	D547Y *	SPD|Mutation Block 2|Motif 6	Disrupted protein function
28	L552F *	SPD|Mutation Block 2|Motif 7	Disrupted protein function
29	M555T *	SPD|Mutation Block 2|Motif 7	Disrupted protein function
30	G559D *	SPD|Mutation Block 2|Motif 7	Disrupted protein function
31	Y562S	Mutation Block 2	KCS2|OCS
32	R569H	Mutation Block 2	KCS2
33	I572del	Mutation Block 2	KCS2
34	K586K	Mutation Block 2	KCS2

* ClinVar Germline Classification: Uncertain significance, Benign, Likely Benign and Conflicting classifications.

## Data Availability

The original contributions presented in this study are included in the article and Supplementary Material. Further inquiries can be directed to the corresponding authors.

## Supplementary Materials

The following supporting information can be downloaded at: https://www.mdpi.com/article/10.3390/ph19030375/s1, Supplementary Data S1: The 65 representative protein sequences of FAM111A that have been used in this study and the corresponding taxonomic groups; Supplementary Data S2: The MSA of FAM111A representative protein sequences; Supplementary Data S3: The phylogenetic tree of FAM111A representative protein sequences; Supplementary Data S4: The list of candidate inhibitors of the FAM111A protein.

## Abbreviations

The following abbreviations are used in this manuscript:

Ad5	Adenovirus 5
AMP	Adenosine monophosphate
APH	Aphidicolin
Arg	Arginine
Asp	Aspartate
BKPyV	BK polyomavirus
CASP7	caspase-7
CFB	complement factor B
cGAS	Cyclic GMP-AMP
circRNA	Circular RNA
CPT	Camptothecin
DBD	dsDNA binding domain
DNA Pol δ	DNA polymerase delta
DP	Dimerization partner
DPCs	DNA protein crosslinks
ds-DNA	Double-stranded DNA
E2F	Adenovirus early gene 2 binding factor
FAM111A	FAM111 trypsin-like peptidase A
FAM111A-DT	FAM111A-divergent transcript
FEN-1	Flap Structure-Specific Endonuclease 1
GCLEB/OCS	Gracile bone dysplasia/Osteocraniostenosis
Glu	Glutamic acid
GMP	Guanosine monophosphate synthase
GOF	gain-of-function
GRKs	G protein receptor kinases
HGFA	Hepatocyte Growth Factor Activator
His	Histidine
HNS CC	Head And Neck Squamous Cell Carcinoma
HR	Host range
HU	Hydroxyurea
IDH	Isocitrate dehydrogenase
IFNs	Interferons
IRF	Interferon Regulatory Factor
JCPyV	JC polyomavirus
KCS2	Kenny-Caffey Syndrome Type 2
KDM3B	Histone lysine demethylase 3B
LINC02550	Long Intergenic Non-Protein Coding RNA 2550
lncRNA	Long noncoding RNA
lncRNA	Linear RNA
LT	Large T antigen
Lys	Lysine
MCPyV	Merkel cell polyomavirus
miRNA	MicroRNA
mRNA	Messenger RNA
MSA	Multiple sequence alignment
MuvB	Multi-vulva class B
NUP62	Nuclear pore glycoprotein p62
PARP	poly(ADP-ribose) polymerase
PARPis	PARP inhibitors
PCNA	Proliferating cell nuclear antigen
PDB	Protein Data Bank
Phe	Phenylalanine
PIP	PCNA interacting peptide
PIPmt	PIP-box Mutations
PRSS16	Thymus-specific serine protease
PSMB8	proteasome subunit beta type-8
PSMB9	Proteasome subunit beta type-9-
RB	Retinoblastoma
RBBP4	RB Binding Protein 4
RCL	Reactive Center Loop
RFC1	Replication factor C 1
RFC3	Replication Factor C3
Ser	Serine
Serpins	SERine Protease INhibitorS
SPD	serine protease domain
SPI-1	Serine Protease Inhibitor 1
SSB	ssDNA- binding protein
ssDNA	Single stranded DNA
STING	Stimulator of Interferon Genes
Thr	Threonin
TOP1	Topoisomerase I
Trp	Tryptophan
Tyr	Tyrosine
UBL	Ubiquitin-Like Domains
UPGMA	Unweighted Pair-Group Method
VACV	Vaccinia virus
Val	Valine
VCAM-1	Vascular Cell Adhesion Molecule 1
YTHDC1	YTH N6-Methyladenosine RNA Binding Protein C1
ZIKV	zika virus
ZNF226	Zinc finger protein 226
